# Associations of weight change with perceived stress and depression: a nationwide population-based study

**DOI:** 10.3389/fpsyt.2026.1941947

**Published:** 2026-09-16

**Authors:** Yujin Bae, Ye-Seul Song, Eun Hee Park, Hyun Ju Hong, Hyewon Kim

**Affiliations:** 1 Department of Psychology, Ewha Womans University, Seoul, Republic of Korea; 2 Big Data Center, Doheon Institute for Digital Innovation in Medicine, Hallym University Medical Center, Anyang, Gyeonggi-do, Republic of Korea; 3 Department of Psychiatry, College of Medicine, Hallym University, Hallym University Sacred Heart Hospital, Anyang, Republic of Korea

**Keywords:** body mass index, depression, population-based study, stress, weight

## Abstract

**Purpose:**

Depression and perceived stress are prevalent mental health problems worldwide, closely associated with physical health. Prior studies have suggested that both weight gain and loss may increase the odds of depression and stress, highlighting the need for clarification using national data.

**Methods:**

Data from the Korea National Health and Nutrition Examination Survey in 2014, 2016, 2018, and 2020 were used. The final analytic sample included 21,599 adults aged ≥19 years. We identified weight change over the past year and categorized it into seven groups (≥10 kg loss, 6–<10 kg loss, 3–<6 kg loss, no weight change, 3–<6 kg gain, 6–<10 kg gain, and ≥10 kg gain). We analyzed the odds of depressive symptoms, self-reported physician diagnosis of depression, and perceived stress according to the change in weight.

**Results:**

Higher odds of depressive symptoms, self-reported physician diagnosis of depression, and perceived stress were observed at both substantial weight loss and weight gain. Compared with participants reporting no weight change, the adjusted odds ratios (95% CIs) for weight loss ≥10 kg were 3.51 (2.30–5.34), 1.99 (1.17–3.78), and 1.83 (1.34–2.48) for depressive symptoms, self-reported physician diagnosis of depression, and perceived stress, respectively. The corresponding estimates for weight gain ≥10 kg were 3.82 (2.72–5.36), 2.80 (1.89–4.14), and 2.28 (1.82–2.85), respectively. No significant interactions were observed between weight change and sex, age, body mass index, or weight control status.

**Conclusions:**

Substantial weight loss and weight gain were associated with elevated odds of adverse mental health outcomes.

## 1 Introduction

Depression and perceived stress are among the most prevalent mental health problems worldwide and represent major public health concerns (1, 2). Across international studies, the prevalence of depressive symptoms ranges from approximately 12–29%, with some longitudinal cohorts reporting lifetime rates up to 37% (3, 4). High levels of perceived stress have also been observed in about 11–23% of adults in vulnerable populations, such as the unemployed (5). In South Korea, nearly one in four adults (23.8%) reports high stress (6). Both depression and perceived stress are associated with impaired functioning, social withdrawal, and reduced quality of life, and constitute major contributors to disability worldwide (7). These burdens underscore the importance of identifying modifiable factors associated with depression and perceived stress.

Although primarily regarded as psychological conditions, depression and stress are closely linked with physical health (8). Prior studies have documented bidirectional associations between changes in weight and mental health. Physical health and mental health are closely interconnected, with longitudinal evidence indicating an association between overweight or obesity and depression (9). Conversely, poor mental health may be associated with weight change through behavioral factors such as reduced physical activity, disrupted sleep, and emotional eating (10, 11), as well as poorer adherence to chronic disease management (12, 13). These reciprocal links are further shaped by sociocultural contexts, particularly in South Korea, where cultural emphasis on physical appearance may amplify the psychological consequences of weight change (14).

Epidemiological evidence supports these links, documenting associations between weight change and depression and suggesting that stress-related processes, such as hypothalamic-pituitary-adrenal (HPA) axis dysregulation and maladaptive eating, may underlie the association (11, 15). Large-scale studies also indicate that excess weight is associated with both clinical and subclinical depression (16), while non-normative weight status, particularly underweight, has also been associated with poorer mental health (17). Nonetheless, much existing evidence is limited to specific populations or suffers from inadequate control of confounders, and large-scale, nationally representative data capturing diverse demographic subgroups remain scarce.

The present study aims to examine associations between weight change and mental health outcomes—depressive symptoms, self-reported physician diagnosis of depression, and perceived stress—using data from the Korea National Health and Nutrition Examination Survey (KNHANES), a nationally representative population-based survey. We further explored whether these associations differed by demographic characteristics, body mass index (BMI), and weight control behavior.

## 2 Methods

### 2.1 Data source

This study used data from the Korea National Health and Nutrition Examination Survey (KNHANES), conducted in 2014, 2016, 2018, and 2020. These survey cycles were selected because the mental health measures required for the present analyses were available in these cycles. KNHANES is a legally mandated, nationally representative survey conducted annually by the KDCA under the National Health Promotion Act (6, 18, 19). The survey collects data on health behaviors, chronic disease prevalence, and dietary and nutritional intake among the Korean population and is used to inform national health policies. The KNHANES employed a rolling sampling design. In 2014 and 2020, the sampling frame was based on the 2010 Population and Housing Census. In 2016 and 2018, it was supplemented with data from the Multi-unit Housing Announced Price database to improve coverage. Data were collected through household interviews and mobile health examination centers and included household demographics, health interviews, physical examinations, and nutrition surveys.

The KNHANES conducted in 2014, 2018, and 2020 were approved by the Institutional Review Board (IRB) of the KDCA. The 2016 survey was exempted from IRB review as it was deemed a government-led public welfare project under the Bioethics and Safety Act.

### 2.2 Subjects

Of the 31,051 participants across the four KNHANES cycles, 24,980 were adults aged 19 years or older. After excluding individuals with incomplete information on relevant variables, 21,599 participants were included in the final analysis (**Figure 1**).

**Figure 1:**
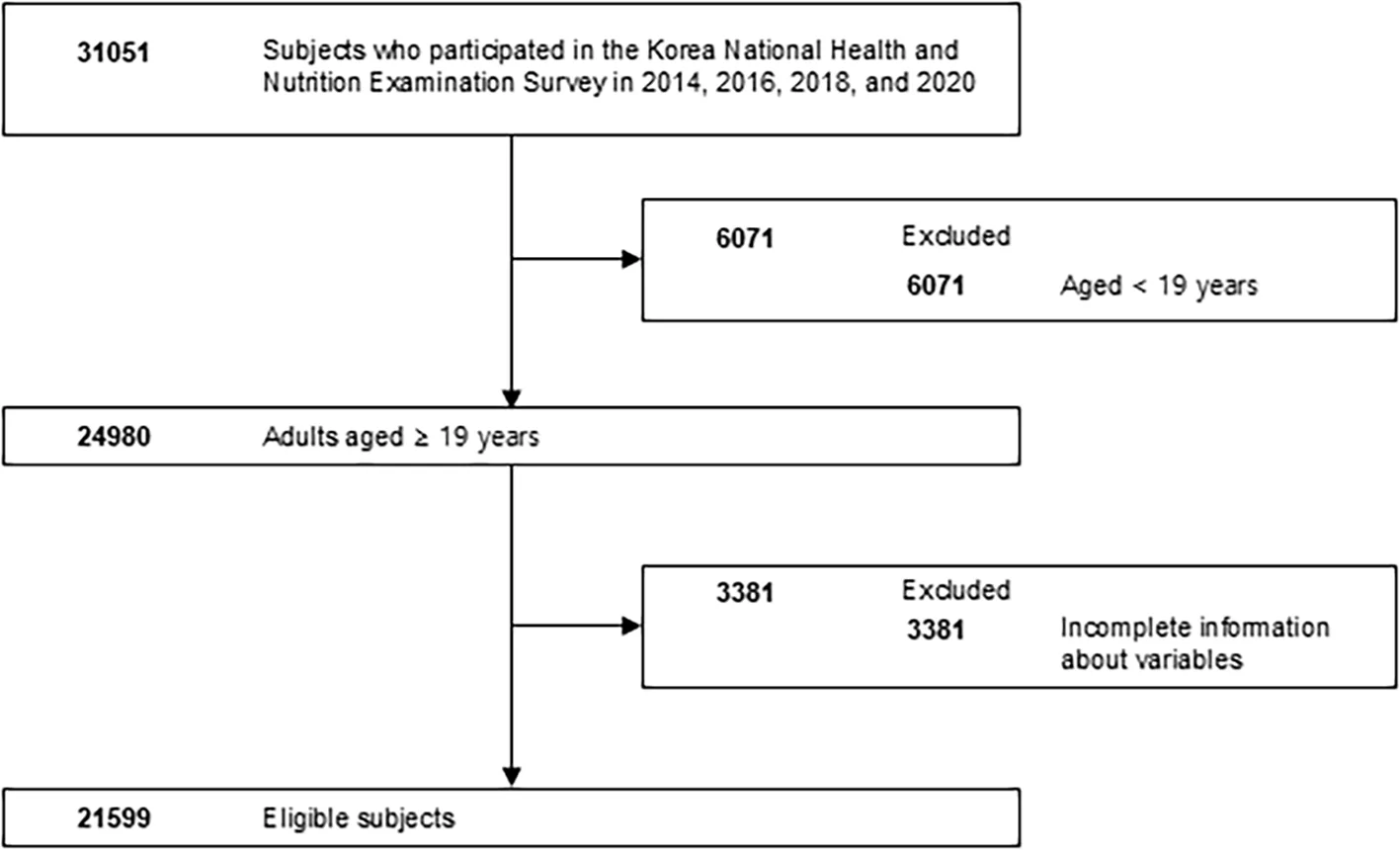
Flowchart showing the selection of subjects for this study.

### 2.3 Weight change

Self-reported weight change over the past year was categorized into seven groups according to the predefined response categories in KNHANES: ≥10 kg loss, 6–<10 kg loss, 3–<6 kg loss, no weight change, 3–<6 kg gain, 6–<10 kg gain, and ≥10 kg gain. No weight change was defined as a change of <3 kg in either direction.

### 2.4 Outcomes

We examined three mental health outcomes: depressive symptoms, self-reported physician diagnosis of depression, and perceived stress. Depressive symptoms were assessed using the Korean version of the Patient Health Questionnaire-9 (PHQ-9), which has demonstrated acceptable reliability and validity in Korean populations (20), a self-administered instrument based on DSM-IV criteria (e.g., “Little interest or pleasure in doing things” over the past two weeks). A score of ≥10 indicated clinically significant symptoms. Self-reported physician diagnosis of depression was based on participants’ report of ever having received a diagnosis of depression from a physician. Perceived stress was measured using a single-item question asking participants to rate their usual level of stress on a 4-point Likert scale (1 = feel very much, 2 = feel much, 3 = feel somewhat, 4 = hardly feel). For the present analysis, responses of 1–2 were classified as high perceived stress and responses of 3–4 as low perceived stress, consistent with categorizations used in large-scale epidemiological research (6). This binary operationalization was used to estimate the odds of high perceived stress within the same logistic-regression framework applied to the other mental health outcomes.

### 2.5 Covariates

Covariates included sex, age, income level, employment status, marital status, living arrangement (living alone), BMI, and weight-control status (based on reported attempts to manage weight in the past year). BMI was calculated from height and weight measured at the time of the KNHANES examination as weight (kg) divided by height squared (m²) and categorized as <18.5, 18.5–<23.0, 23.0–<25.0, 25.0–<30.0, and ≥30.0 kg/m², according to the Korean Society for the Study of Obesity guidelines (21).

### 2.6 Statistical analysis

Participants with missing data on variables required for the primary analysis were excluded using complete-case analysis. Of 24,980 eligible adults, 21,599 (86.5%) were retained in the final analytic sample, while 3,381 (13.5%) were excluded because of missing data on one or more required variables. Missingness by individual study variable ranged from 0% to 12.02%. Variable-specific missingness and comparisons of characteristics between participants included in and excluded from the complete-case analysis are presented in **Supplementary Tables S1, S2**, respectively.

Descriptive statistics were reported as frequencies and weighted proportions, as all variables were categorical. Logistic regression analyses were used to examine associations between weight change and mental health outcomes. Odds ratios (ORs) and 95% confidence intervals (CIs) were calculated. Two models were tested, where Model 1 was unadjusted and Model 2 was adjusted for sex, age, income level, employment status, marital status, living arrangement (living alone). To ensure valid population-level estimates, all analyses accounted for the complex sampling design of the KNHANES, including stratification, clustering, and sampling weights, as recommended by the Korea Disease Control and Prevention (18, 19). Weights were adjusted for sampling probability, non-response, and post-stratification. Integrated weights across the four KNHANES cycles were applied. All analyses were conducted using Stata 18.0 (22), with statistical significance set at *p* < 0.05. Subgroup analyses by sex, age, BMI, and weight-control status were considered exploratory, with potential effect modification evaluated primarily using formal interaction tests.

Three sets of sensitivity analyses were conducted to assess the robustness of the findings. First, to examine the robustness of the findings to the inclusion of BMI and weight-control status, four model specifications were compared: Model A corresponded to the primary adjusted model; Model B additionally included BMI; Model C additionally included weight-control status; and Model D included both BMI and weight-control status. Second, to assess potential residual confounding, additional sensitivity analyses were conducted by further adjusting the fully adjusted model for diabetes, hypertension, thyroid disease, smoking, alcohol use, activity limitation, and education level. Third, to account for potential secular changes across survey cycles, sensitivity analyses were conducted by additionally adjusting for survey year as a categorical variable. Given the unique circumstances of the COVID-19 pandemic, the analyses were also repeated after excluding the 2020 survey cycle.

## 3 Results

### 3.1 Characteristics of subjects

**Table 1** summarizes the characteristics of the study population. Women made up 50.1% of the total sample. The largest proportion of participants was aged 36–64 years (55.9%), followed by those aged 19–35 years (28.6%) and 65 years or older (15.6%). Income was evenly distributed across quartiles, with each group comprising about 25% of the total sample. Most participants were employed (64.4%), and 66.1% were married. A small proportion reported living alone (9.8%). Regarding mental health, 5.4% had significant depressive symptoms (PHQ-9 ≥10). High perceived stress was reported by 27.3%, and 4.6% reported having received a physician diagnosis of depression. In terms of BMI, 4.2% were underweight (<18.5 kg/m²), 49.0% fell within the normal range (18.5–<23 kg/m²), and 3.5% had a BMI of ≥30 kg/m². Over the past year, 66.3% of participants reported attempting to control their weight. In terms of actual weight change, 60.8% maintained a stable weight (–3 to 3 kg). The remainder experienced various degrees of weight gain or loss, with 1.2% reporting a loss of ≥10 kg and 2.4% reporting a gain of ≥10 kg.

**Table 1:** Characteristics of study subjects (N = 21,599).

Characteristics	N (Weighted %)
Sex	
Male	9,428 (49.9)
Female	12,171 (50.1)
Age group	
19–35 years	4,526 (28.6)
36–64 years	11,689 (55.9)
≥65 years	5,384 (15.6)
Income quartile	
Low	5,232 (24.9)
Middle-low	5,433 (25.1)
Middle-high	5,457 (25.1)
High	5,477 (25.0)
Employment status	
Employed	13,087 (64.4)
Unemployed	8,512 (35.6)
Marital status	
Married	15,069 (66.1)
Divorced	951 (3.9)
Bereaved	1,781 (5.8)
Unmarried	3,794 (24.2)
Others	4 (0.0)
Living alone	2,500 (9.8)
Depressive symptoms	
Yes (PHQ-9 score ≥10)	1,228 (5.4)
No (PHQ-9 score <10)	20,371 (94.6)
Perceived stress	
High	5,627 (27.3)
Low	15,972 (72.7)
Self-reported physician diagnosis of depression	1,069 (4.6)
BMI (kg/m^2^)	
<18.5	847 (4.2)
18.5–23	10,695 (49.0)
23–25	6,019 (27.7)
25–30	3,348 (15.8)
≥30	690 (3.5)
Weight control status (past 12 months)	
Engaged	14,022 (66.3)
Not engaged	7,577 (33.7)
Weight change (kg)	
≥10 kg loss	237 (1.2)
6–<10 kg loss	481 (2.5)
3–<6 kg loss	2,142 (10.0)
No weight change	13,659 (60.8)
3–<6 kg gain	3,765 (18.2)
6–<10 kg gain	895 (4.9)
≥10 kg gain	420 (2.4)

PHQ-9, Patient Health Questionnaire-9.

### 3.2 Associations of weight change with depressive symptoms, self-reported physician diagnosis of depression, and perceived stress

**Table 2** and **Figure 2** present the adjusted ORs and 95% CIs for depressive symptoms, self-reported physician diagnosis of depression, and perceived stress according to weight change categories. Compared with individuals whose weight remained stable (–3 to 3 kg), both weight loss and weight gain were generally associated with poorer mental health outcomes.

**Table 2:** Odds ratios and 95% confidence intervals of weight change on depressive symptoms, self-reported physician diagnosis of depression, and perceived stress (*N* = 21,599).

Weight change (kg)	Depressive symptoms		self-reported physician diagnosis of depression			Perceived stress	
	Crude	Adjusted	Crude	Adjusted		Crude	Adjusted
	Odds ratio (95% confidence intervals)						
≥10 kg loss	4.09 (2.75–6.07)^***^	3.51 (2.30–5.34)^***^	1.95 (1.16–3.27)^*^		1.99 (1.17–3.78)^*^	2.08 (1.54–2.82)^***^	1.83 (1.34–2.48)^***^
6–<10 kg loss	1.65 (1.09–2.53)^*^	1.57 (1.01–2.42)^*^	1.88 (1.15–3.07)^*^		2.07 (1.23–3.47)^**^	1.51 (1.21–1.88)^***^	1.37 (1.09–1.72)^**^
3–<6 kg loss	1.85 (1.48–2.31)^***^	1.72 (1.37–2.16)^***^	1.25 (0.97–1.59)		1.23 (0.96–1.58)	1.42 (1.26–1.60)^***^	1.38 (1.22–1.56)^***^
No weight change	1 (Ref.)	1 (Ref.)	1 (Ref.)		1 (Ref.)	1 (Ref.)	1 (Ref.)
3–<6 kg gain	1.43 (1.18–1.73)^***^	1.36 (1.12–1.65)^**^	1.51 (1.24–1.83)^***^		1.50 (1.23–1.83)^***^	1.44 (1.31–1.59)^***^	1.28 (1.16–1.41)^***^
6–<10 kg gain	2.43 (1.85–3.17)^***^	2.27 (1.70–3.03)^***^	1.73 (1.24–2.42)^**^		1.97 (1.39–2.79)^***^	1.85 (1.58–2.16)^***^	1.52 (1.29–1.78)^***^
≥10 kg gain	4.51 (3.29–6.18)^***^	3.82 (2.72–5.36)^***^	2.66 (1.84–3.85)^***^		2.80 (1.89–4.14)^***^	2.84 (2.29–3.52)^***^	2.28 (1.82–2.85)^***^

Adjusted for sex, age, income, employment status, marital status, living alone.

^*^ *P* < 0.05, ^**^P < 0.01, ^***^P < 0.001.

**Figure 2:**
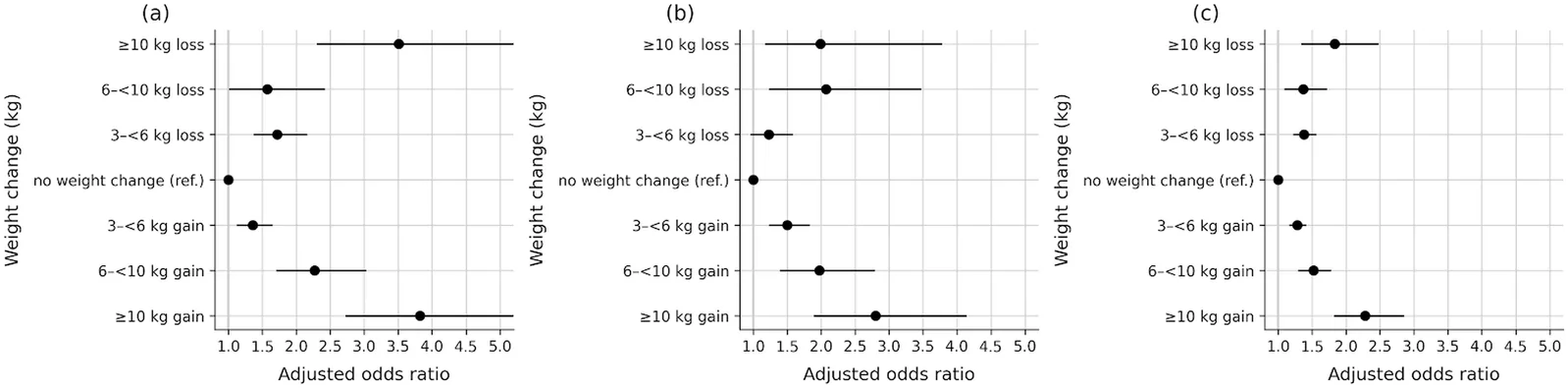
Forest plot for adjusted odds ratios and 95% confidence intervals by weight changes. Forest plot showing the adjusted odds ratios and corresponding 95% confidence intervals for depressive symptoms (PHQ-9 ≥10), self-reported physician diagnosis of depression and perceived stress according to weight change categories. Adjusted for sex, age, income, employment status, marital status, living alone. **(A)** Depressive symptoms; **(B)** self-reported physician diagnosis of depression; **(C)** Perceived stress.

The likelihood of reporting significant depressive symptoms was generally higher with greater weight gain, reaching the highest odds among those who gained ≥10 kg (adjusted OR [aOR] = 3.82, 95% CI: 2.72–5.36, *p* < 0.001). In addition, weight loss of ≥10 kg was also strongly associated with depressive symptoms (aOR = 3.51, 95% CI: 2.30–5.34, *p* < 0.001), while more moderate weight loss (–6 to –3 kg) showed a smaller but still significant increase in the odds (aOR = 1.72, 95% CI: 1.37–2.16, *p* < 0.001). For self-reported physician diagnosis of depression, significant associations were observed for most categories of weight change in both directions. However, moderate weight loss (–6 to –3 kg) was not significantly related to self-reported physician diagnosis of depression (aOR = 1.23, 95% CI: 0.96–1.58). For perceived stress, both weight loss and weight gain were associated with higher odds of perceived stress, with generally stronger associations observed at larger magnitudes of weight change. The strongest associations were seen for weight loss of ≥10 kg (aOR = 1.83, 95% CI: 1.34–2.48, *p* < 0.001) and weight gain of ≥10 kg (aOR = 2.28, 95% CI: 1.82–2.85, *p* < 0.001).

### 3.3 Subgroup analyses

**Table 3** presents the associations between weight change and depressive symptoms, self-reported physician diagnosis of depression, and perceived stress across subgroups defined by weight-control status, sex, age, and BMI. No statistically significant interactions were observed in the adjusted analyses for any of the subgroup variables or mental health outcomes (all *p* for interaction >.05). In the crude analyses, significant interactions by age were observed for depressive symptoms and perceived stress (*p* for interaction = 0.008 and 0.021, respectively); however, these interactions were no longer statistically significant after adjustment.

**Table 3:** Odds ratios and 95% confidence intervals of weight change on depressive symptoms, self-reported physician diagnosis of depression, and perceived stress according to subgroups.

Subgroup		Weight change (kg)	Depressive symptoms		Self-reported physician diagnosis of depression		Perceived stress	
			Crude	Adjusted	Crude	Adjusted	Crude	Adjusted
			Odds ratio (95% confidence intervals)					
Weight control status (past 12 months)								
Not engaged	≥10 kg loss		8.40 (3.98–17.75)^***^	9.03 (4.03–20.25)^***^	1.20 (0.36–4.03)	1.24 (0.36–4.30)	4.33 (2.23–8.40)^***^	4.19 (2.20–7.94)^***^
	6–<10 kg loss		1.82 (0.90–3.70)	1.62 (0.82–3.23)	3.65 (1.51–8.80)^**^	3.67 (1.40–9.62) ^**^	1.51 (0.92–2.47)	1.52 (0.92–2.51)
	3–<6 kg loss		2.36 (1.70–3.28)^***^	2.18 (1.55–3.06)^***^	1.26 (0.84–1.89)	1.15 (0.76–1.75)	1.46 (1.17–1.82)^**^	1.50 (1.20–1.88) ^***^
	No weight change		1 (Ref.)	1 (Ref.)	1 (Ref.)	1 (Ref.)	1 (Ref.)	1 (Ref.)
	3–<6 kg gain		1.48 (1.08–2.04)^*^	1.48 (1.06–2.07)^*^	1.29 (0.91–1.85)	1.31 (0.92–1.86)	1.67 (1.41–1.98)^***^	1.50 (1.26–1.78)^***^
	6–<10 kg gain		3.19 (2.07–4.92)^***^	3.47 (2.17–5.53)^***^	1.21 (0.64–2.29)	1.41 (0.75–2.67)	2.05 (1.55–2.72)^***^	1.70 (1.27–2.26)^***^
	≥10 kg gain		4.20 (2.23–7.92)^***^	4.43 (2.26–8.72)^***^	3.48 (1.75–6.91)^***^	4.35 (2.13–8.88)^***^	3.18 (2.04–4.96)^***^	2.63 (1.67–4.16)^***^
Engaged	≥10 kg loss		3.35 (2.09–5.38)^***^	2.54 (1.54–4.19)^***^	2.17 (1.22–3.84)^***^	2.23 (1.24–4.02)^**^	1.72 (1.22–2.41)^**^	1.48 (1.04–2.10)^*^
	6–<10 kg loss		1.66 (0.98–2.83)	1.58 (0.90–2.76)	1.35 (0.79–2.31)	1.53 (0.87–2.67)	1.49 (1.15–1.92)^**^	1.33 (1.02–1.73)^*^
	3–<6 kg loss		1.68 (1.26–2.24)^***^	1.54 (1.14–2.09)^**^	1.25 (0.93–1.70)	1.29 (0.95–1.74)	1.39 (1.21–1.61)^***^	1.33 (1.15–1.55)^***^
	No weight change		1 (Ref.)	1 (Ref.)	1 (Ref.)	1 (Ref.)	1 (Ref.)	1 (Ref.)
	3–<6 kg gain		1.46 (1.15–1.86)^*^	1.33 (1.05–1.69)^*^	1.60 (1.27–2.01)^***^	1.58 (1.25–2.00)^***^	1.36 (1.20–1.53)^***^	1.20 (1.07–1.36)^**^
	6–<10 kg gain		2.16 (1.54–3.04)^***^	1.83 (1.28–2.64) ^**^	1.98 (1.35–2.89)^***^	2.21 (1.48–3.29)^***^	1.76 (1.45–2.12)^***^	1.44 (1.19–1.75)^***^
	≥10 kg gain		4.80 (3.26–7.06)^***^	3.65 (2.42–5.51)^***^	2.39 (1.54–3.72)^***^	2.37 (1.48–3.80)^***^	2.70 (2.09–3.48)^***^	2.16 (1.66–2.81)^***^
*P* for interaction			0.232	0.131	0.210	0.272	0.133	0.107
Sex								
Male	≥10 kg loss		4.59 (2.44–8.60)^***^	3.57 (1.88–6.78)^***^	4.48 (2.16–9.27)^***^	3.66 (1.76–7.58)^**^	1.80 (1.16–2.80)^**^	1.70 (1.08–2.67)^*^
	6–<10 kg loss		1.67 (0.82–3.39)	1.52 (0.75–3.08)	2.15 (0.74–6.24)	2.08 (0.72–6.05)	1.20 (0.86–1.68)	1.10 (0.78–1.55)
	3–<6 kg loss		1.97 (1.35–2.88)^***^	1.77 (1.19–2.63)^**^	1.23 (0.74–2.06)	1.11 (0.66–1.87)	1.35 (1.12–1.62)^**^	1.34 (1.11–1.62)^**^
	No weight change		1 (Ref.)	1 (Ref.)	1 (Ref.)	1 (Ref.)	1 (Ref.)	1 (Ref.)
	3–<6 kg gain		1.35 (0.96–1.90)	1.30 (0.91–1.86)	1.38 (0.90–2.10)	1.38 (0.90–2.12)	1.36 (1.17–1.58)^***^	1.23 (1.06–1.44)^**^
	6–<10 kg gain		2.12 (1.34–3.33)^**^	1.93 (1.14–3.28)^*^	1.50 (0.78–2.85)	1.47 (0.72–3.00)	1.61 (1.27–2.05)^***^	1.37 (1.07–1.76)^*^
	≥10 kg gain		4.80 (2.69–8.58)^***^	4.41 (2.29–8.50)^***^	4.00 (2.13–7.50)^***^	3.83 (1.94–7.56)^***^	2.50 (1.76–3.54)^***^	2.12 (1.48–3.04)^***^
Female	≥10 kg loss		3.90 (2.34–6.51)^***^	3.31 (1.91–5.73)^***^	1.00 (0.50–2.01)	1.07 (0.54–2.13)	2.41 (1.60–3.64)^***^	2.04 (1.34–3.13)^**^
	6–<10 kg loss		1.78 (1.04–3.04)^*^	1.57 (0.90–2.76)	1.94 (1.19–3.16)^**^	2.04 (1.22–3.38)^**^	1.99 (1.45–2.71)^***^	1.73 (1.26–2.38)^**^
	3–<6 kg loss		1.81 (1.40–2.34)^***^	1.66 (1.28–2.15)^***^	1.28 (0.97–1.70)	1.29 (0.97–1.71)	1.50 (1.29–1.75)^***^	1.41 (1.21–1.65)^***^
	No weight change		1 (Ref.)	1 (Ref.)	1 (Ref.)	1 (Ref.)	1 (Ref.)	1 (Ref.)
	3–<6 kg gain		1.36 (1.08–1.72)^**^	1.43 (1.13–1.80)^**^	1.40 (1.13–1.75)^**^	1.53 (1.23–1.91)^***^	1.48 (1.32–1.67)^***^	1.36 (1.21–1.53)^***^
	6–<10 kg gain		2.72 (1.97–3.74)^***^	2.67 (1.91–3.72)^***^	1.92 (1.31–2.80)^**^	2.20 (1.48–3.27)^***^	2.15 (1.74–2.65)^***^	1.78 (1.44–2.22)^***^
	≥10 kg gain		4.40 (3.02–6.39)^***^	3.83 (2.60–5.63)^***^	2.15 (1.36–3.41)^**^	2.28 (1.43–3.67)^**^	3.22 (2.46–4.22)^***^	2.65 (2.01–3.49)^***^
P for interaction			0.965	0.801	0.077	0.104	0.208	0.129
19–35 years	≥10 kg loss		2.14 (1.07–4.31)^*^	2.28 (1.09–4.77)^*^	1.68 (0.64–4.44)	1.76 (0.63–4.88)	1.30 (0.84–2.00)	1.37 (0.89–2.11)
	6–<10 kg loss		0.98 (0.43–2.22)	1.02 (0.44–2.34)	2.21 (0.72–6.80)	2.35 (0.75–7.33)	1.07 (0.74–1.55)	1.11 (0.77–1.60)
	3–<6 kg loss		1.62 (1.02–2.57)^*^	1.64 (1.03–2.61)^*^	1.65 (0.94–2.91)	1.70 (0.96–2.99)	1.22 (0.97–1.54)	1.23 (0.98–1.55)
	No weight change		1 (Ref.)	1 (Ref.)	1 (Ref.)	1 (Ref.)	1 (Ref.)	1 (Ref.)
	3–<6 kg gain		1.54 (1.08–2.19)^*^	1.57 (1.10–2.24)^*^	1.99 (1.26–3.14)^**^	1.96 (1.25–3.08)^**^	1.17 (0.99–1.39)	1.17 (0.98–1.39)
	6–<10 kg gain		1.75 (1.13–2.70)^*^	1.94 (1.23–3.06)^**^	2.25 (1.27–3.96)^**^	2.41 (1.34–4.35)^**^	1.20 (0.94–1.52)	1.25 (0.98–1.60)
	≥10 kg gain		3.31 (2.11–5.20)^***^	3.55 (2.25–5.60)^***^	2.46 (1.34–4.53)^**^	2.66 (1.43–4.96)^**^	1.80 (1.33–2.45)^***^	1.92 (1.42–2.60)^***^
36–64 years	≥10 kg loss		6.79 (3.62–12.75)^***^	4.54 (2.49–8.28)^***^	2.98 (1.31–6.75)^**^	2.23 (1.00–5.00)	2.65 (1.64–4.26)^***^	2.50 (1.55–4.04)^***^
	6–<10 kg loss		1.79 (0.83–3.85)	1.78 (0.82–3.86)	1.95 (1.07–3.56)^*^	2.17 (1.15–4.11)^*^	1.46 (1.03–2.07)^*^	1.42 (1.00–2.02)^*^
	3–<6 kg loss		1.83 (1.33–2.52)^***^	1.71 (1.23–2.38)^**^	1.21 (0.85–1.74)	1.21 (0.84–1.73)	1.39 (1.18–1.65)^***^	1.38 (1.16–1.63)^***^
	No weight change		1 (Ref.)	1 (Ref.)	1 (Ref.)	1 (Ref.)	1 (Ref.)	1 (Ref.)
	3–<6 kg gain		1.29 (0.98–1.71)	1.20 (0.90–1.59)	1.67 (1.30–2.14)^***^	1.45 (1.12–1.87)^**^	1.37 (1.21–1.55)^***^	1.37 (1.20 -1.55)^***^
	6–<10 kg gain		3.58 (2.48–5.16)^***^	3.26 (2.22–4.79)^***^	2.14 (1.43–3.18)^***^	1.80 (1.18–2.75)^**^	1.91 (1.52–2.39)^***^	1.88 (1.50–2.36)^***^
	≥10 kg gain		6.62 (4.00–10.94)^***^	4.71 (2.69–8.22)^***^	5.10 (3.09–8.41)^***^	3.37 (1.94–5.87)^***^	3.16 (2.24–4.47)^***^	3.10 (2.18–4.39)^***^
≥ 65 years	≥10 kg loss		5.97 (2.91–12.26)^***^	5.67 (2.53–12.69)^***^	2.48 (0.99–6.20)	2.27 (0.92–5.61)	2.59 (1.30–5.18)^**^	2.62 (1.28–5.37)^**^
	6–<10 kg loss		2.87 (1.55–5.33)^**^	2.72 (1.42–5.21)^**^	2.10 (0.97–4.54)	1.92 (0.90–4.09)	2.27 (1.41–3.65)^**^	2.08 (1.29–3.37) ^**^
	3–<6 kg loss		1.91 (1.42–2.58)^***^	1.73 (1.26–2.37)^**^	1.11 (0.76–1.61)	1.06 (0.72–1.57)	1.66 (1.31–2.10)^***^	1.63 (1.28–2.08)^***^
	No weight change		1 (Ref.)	1 (Ref.)	1 (Ref.)	1 (Ref.)	1 (Ref.)	1 (Ref.)
	3–<6 kg gain		1.52 (1.04–2.23)^*^	1.37 (0.93–2.04)	1.16 (0.78–1.72)	1.05 (0.71–1.57)	1.23 (0.93–1.63)	1.16 (0.88–1.15)
	6–<10 kg gain		0.57 (0.14–2.41)	0.50 (0.12–2.08)	1.64 (0.33–8.16)	1.54 (0.33–7.28)	2.10 (0.94–4.66)	1.96 (0.88–4.39)
	≥10 kg gain		2.55 (0.69–9.46)	2.16 (0.53–8.90)	2.30 (0.62–8.58)	2.03 (0.48–8.51)	1.97 (0.69–5.64)	1.94 (0.67–5.63)
*P* for interaction			0.008	0.066	0.667	0.836	0.021	0.094
Body mass index (kg/m^2^)								
<18.5	≥10 kg loss		11.68(2.79–48.82)^**^	11.56 (2.13–62.84)^**^	8.19 (1.94–34.56)^**^	12.44 (3.68–42.10) ^***^	1.92 (0.48–7.73)	2.75 (0.63–12.08)
	6–<10 kg loss		1.71 (0.31–9.25)	1.70 (0.28–10.35)	0.87 (0.13–5.82)	0.67 (0.13–3.58)	2.12 (0.82–5.52)	2.72 (1.05–7.01)
	3–<6 kg loss		2.01 (0.35–4.13)	2.24 (1.08–4.66)^*^	0.92 (0.30–2.77)	1.22 (0.37–4.00)	1.80 (1.04–3.12)^*^	1.91 (1.08–3.38)^*^
	No weight change		1 (Ref.)	1 (Ref.)	1 (Ref.)	1 (Ref.)	1 (Ref.)	1 (Ref.)
	3–<6 kg gain		3.69 (1.49–9.11)^*^	3.59 (1.47–8.79)^**^	5.12 (1.85–14.22)^**^	6.01 (2.08–17.35)^**^	1.45 (0.70–3.00)	1.22 (0.59–2.53)
	6–<10 kg gain		1	1	1	1	23.59 (1.44–386.18)^*^	19.41 (1.12–336.39)^*^
	≥10 kg gain		–	–	–	–	-	–
18.5–23	≥10 kg loss		4.42 (2.51–7.77)^***^	3.98 (2.22–7.13)^***^	1.34 (0.53–3.39)	1.13 (0.52–3.30)	2.47 (1.54–3.95)^***^	2.28 (1.14–3.71)^***^
	6–<10 kg loss		1.82 (1.05–3.16)^*^	1.70 (0.97–2.98)	2.08 (1.18–3.68)^*^	2.35 (1.27–4.33)^**^	1.45 (1.06–1.98)^*^	1.35 (0.98–1.85)
	3–<6 kg loss		1.61 (1.21–2.16)^**^	1.53 (1.14–2.07)^**^	1.45 (1.04–2.03)^*^	1.50 (1.07–2.10)^*^	1.36 (1.16–1.60)^***^	1.34 (1.13–1.58)^**^
	No weight change		1 (Ref.)	1 (Ref.)	1 (Ref.)	1 (Ref.)	1 (Ref.)	1 (Ref.)
	3–<6 kg gain		1.49 (1.13–1.97)^**^	1.45 (1.10–1.91)^**^	1.63 (1.22–2.17)^**^	1.69 (1.26–2.26)^***^	1.45 (1.26–1.66)^***^	1.23 (1.07–1.42)^**^
	6–<10 kg gain		3.39 (2.10–5.48)^***^	3.25 (1.95–5.43)^***^	1.91 (1.04–3.51)^*^	2.27 (1.20–4.30)^*^	1.54 (1.11–2.14)^**^	1.22 (0.87–1.71)
	≥10 kg gain		7.14 (3.41–14.94)^***^	5.60 (2.53–12.39)^***^	4.61 (2.00–10.63)^***^	4.04 (1.64–9.98)^**^	2.51 (1.43–4.40)^**^	1.99 (1.13 – 3.51)^**^
23–25	≥10 kg loss		4.20 (1.83–9.61)^**^	4.00 (1.57–10.18)^**^	2.50 (0.95–6.58)	2.58 (0.92–7.23)	2.11 (1.10–4.07)^*^	1.68 (0.84–3.35)
	6–<10 kg loss		1.19 (0.43–3.35)	1.10 (0.40–3.07)	1.57 (0.45–5.47)	1.63 (0.46–5.74)	1.35 (0.84–2.19)	1.17 (0.73–1.89)
	3–<6 kg loss		1.73 (1.11–2.70)^*^	1.47 (0.91–2.37)	1.02 (0.62–1.67)	0.94 (0.58–1.54)	1.33 (1.05–1.68)^*^	1.24 (0.97–1.59)
	No weight change		1 (Ref.)	1 (Ref.)	1 (Ref.)	1 (Ref.)	1 (Ref.)	1 (Ref.)
	3–<6 kg gain		1.51 (1.05–2.16)^*^	1.35 (0.92–1.98)	1.28 (0.90–1.82)	1.17 (0.83–1.65)	1.40 (1.18–1.67)^***^	1.23 (1.03–1.47)^*^
	6–<10 kg gain		2.33 (1.48–3.65)^***^	1.92 (1.14–3.23)^*^	1.54 (0.93–2.56)	1.47 (0.84–2.56)	2.11 (1.60–2.77)^***^	1.68 (1.27–2.24)^***^
	≥10 kg gain		3.77 (2.19–6.47)^***^	2.76 (1.49–5.13)^**^	2.38 (1.38–4.10)^**^	2.17 (1.20–3.94)^*^	2.50 (1.77–3.52)^***^	1.95 (1.36–2.79)^***^
25–30	≥10 kg loss		3.33 (0.93–11.93)	1.98 (0.50–7.94)	0.59 (0.08–4.45)	0.49 (0.06–4.12)	2.06 (1.09–3.87)^*^	1.45 (0.75–2.80)
	6–<10 kg loss		3.10 (1.16–8.30)^*^	2.88 (1.08–7.69^*^	2.82 (0.93–8.55)	3.19 (1.04–9.82)^*^	1.66 (0.93–2.99)	1.35 (0.74–2.45)
	3–<6 kg loss		3.66 (1.88–7.14)^***^	3.01 (1.54–5.88)^**^	1.08 (0.52–2.23)	0.92 (0.43–1.93)	1.74 (1.23–2.45) ^**^	1.62 (1.14–2.31)^**^
	No weight change		1 (Ref.)	1 (Ref.)	1 (Ref.)	1 (Ref.)	1 (Ref.)	1 (Ref.)
	3–<6 kg gain		1.50 (0.92–2.46)	1.28 (0.77–2.11)	1.64 (1.07–2.53)^*^	1.58 (0.99–2.54)	1.60 (1.26–2.03) ^***^	1.38 (1.08–1.77)^*^
	6–<10 kg gain		3.40 (1.87–6.18)^***^	2.53 (1.25–5.12)^*^	2.17 (1.07–4.42)^*^	2.18 (1.02–4.66)^*^	1.97 (1.46–2.66) ^***^	1.41 (1.02–1.94)^*^
	≥10 kg gain		7.66 (3.69–15.93)^***^	5.33 (2.14–13.30)^***^	3.82 (1.74–8.39)^**^	3.40 (1.22–9.43)^*^	4.21 (2.79–6.35) ^***^	2.93 (1.89–4.55)^***^
≥30	≥10 kg loss		1.75 (0.33–9.19)	1.34 (0.22–8.06)	3.03 (0.63–14.53)	4.27 (0.77–23.78)	0.82 (0.27–2.46)	0.94 (0.33–2.73)
	6–<10 kg loss		0.31 (0.04–2.63)	0.27 (0.03–2.50)	1.31 (0.26–6.64)	1.29 (0.18–9.06)	1.93 (0.66–5.68)	2.12 (0.69–6.54)
	3–<6 kg loss		1.81 (0.59–5.61)	1.49 (0.43–5.19)	1.20 (0.31–4.64)	0.92 (0.22–3.94)	1.48 (0.76–2.91)	1.40 (0.71–2.78)
	No weight change		1 (Ref.)	1 (Ref.)	1 (Ref.)	1 (Ref.)	1 (Ref.)	1 (Ref.)
	3–<6 kg gain		0.96 (0.31–2.96)	0.98 (0.32–2.96)	1.04 (0.33–3.27)	1.12 (0.36–3.47)	1.72 (1.04–2.85)^*^	1.71 (1.01–2.89)^*^
	6–<10 kg gain		1.45 (0.58–3.59)	1.44 (0.53–3.88)	1.70 (0.58–5.00)	1.94 (0.56–6.81)	1.99 (1.11–3.56)^*^	1.96 (1.06–3.62)^*^
	≥10 kg gain		3.98 (1.65–9.60)^**^	3.70 (1.50–9.17)^**^	1.31 (0.40–4.26)	1.19 (0.29–4.94)	2.82 (1.51–5.29)^**^	2.63 (1.38–5.04)^**^
*P* for interaction			0.4553	0.5139	0.5037	0.3735	0.7095	0.7991

Adjusted for sex, age, income, employment status, marital status, living alone, body mass index and weight control status (past 12 months).

^*^ P<0.05, ^**^P <0.01, ^***^P <0.001.

### 3.4 Sensitivity analyses

Sensitivity analyses showed that the associations between weight change and the three mental health outcomes remained highly consistent across models with and without adjustment for BMI and weight-control status, with only minimal changes in the OR estimates and no changes in the overall pattern of statistical significance (**Supplementary Table S3**). In the additional sensitivity analysis, further adjustment for diabetes, hypertension, thyroid disease, smoking, alcohol use, activity limitation, and education level attenuated some associations, but the overall pattern of findings remained generally consistent. Most associations with depressive symptoms remained statistically significant, although the association between weight loss of 6 to <10 kg and depressive symptoms was attenuated and was no longer statistically significant (**Supplementary Table S4**).

Additional adjustment for survey year resulted in minimal changes in the associations between weight change and the three mental health outcomes. When the 2020 survey cycle was excluded, the overall pattern remained generally consistent, although the association between weight loss of ≥10 kg and self-reported physician diagnosis of depression was attenuated and was no longer statistically significant (**Supplementary Table S5**).

## 4 Discussion

This study demonstrated associations of weight change during the past year with depressive symptoms, self-reported physician diagnosis of depression, and perceived stress using nationally representative data. As a result, three major findings were identified. First, compared to stable weight, both weight gain and weight loss were associated with poorer mental health outcomes, showing an approximately U-shaped pattern. Second, weight change was more strongly associated with depressive symptoms measured by the PHQ-9 scores than with self-reported physician diagnosis of depression, suggesting that weight change is linked with psychological distress even at subclinical levels. Third, there were no significant interaction effects of sex, age, BMI, and weight control efforts with weight changes on the mental health outcomes.

In our study, weight gain showed pronounced associations with adverse mental health outcomes. Previous studies have reported that obesity is associated with mental health problems such as depression (16, 23), and the shared biological pathways (e.g., HPA dysregulation, neuroinflammation, and leptin/insulin dysregulation) have been suggested to underlie this association (11). However, our study found that even among individuals with normal BMI, weight gain remained associated with poorer mental health outcomes. Prior studies have suggested that body dissatisfaction mediates the relationship between weight gain and depression, particularly among women, and that sociocultural norms and weight stigma may be associated with greater emotional distress and internalized shame (24, 25). Conversely, mental health problems may also be associated with subsequent weight gain through atypical depressive symptoms such as increased appetite, emotional eating, binge episodes, and stress-related neuroendocrine changes that elevate cortisol levels and favor fat storage (10, 11). These potential pathways highlight the complex and potentially bidirectional association between weight gain and psychological vulnerability.

Weight loss was likewise associated with poorer mental health. Excessive or illness-related weight reduction may co-occur with fatigue, loss of vitality, and greater vulnerability to stress, all of which are associated with poorer psychological well-being (26, 27). At the same time, depressive symptoms are often accompanied by appetite suppression, reduced motivation to eat, and psychomotor changes, which may be associated with subsequent weight loss. Body image dissatisfaction is also associated with intentional weight-loss behaviors and psychological vulnerability, including depression (28, 29). These findings are consistent with a potentially bidirectional relationship between weight loss and psychological distress; however, the direction of these associations cannot be determined from the present cross-sectional data.

The stronger associations observed for depressive symptoms than for self-reported physician diagnosis of depression may have several possible explanations. One possibility is that individuals with clinically significant depressive symptoms who have not received a physician diagnosis are included in the non-diagnosed group, which could attenuate associations with self-reported physician diagnosis (2, 30). Treatment or help-seeking among individuals who have received a diagnosis could also influence their current symptom levels. However, these mechanisms were not directly examined in the present study. Other factors, including differences in measurement timeframes, healthcare access, recall and diagnostic practices, and the distinction between current depressive symptoms and a history of physician diagnosis, may also contribute to the observed differences between the two outcomes. Accordingly, these explanations should be regarded as hypotheses rather than established mechanisms.

We found no strong statistical evidence of effect modification by sex, age, BMI, or weight-control status in the adjusted analyses. However, several subgroup-specific estimates were imprecise, particularly in strata with small sample sizes. These exploratory findings should therefore be interpreted cautiously and warrant confirmation in studies with sufficient power to examine potential effect modification.

To our knowledge, this is one of the first studies in South Korea to focus on weight change rather than static weight status, while integrating both self-reported symptoms and self-reported physician diagnosis of depression. Stratified analyses by sex, age, BMI, and weight-control status further enabled the exploration of effect modification. Nevertheless, several limitations should be considered.

First, depression and stress were assessed by self-report, which may introduce reporting bias. The cross-sectional design precludes causal inference, leaving the direction of associations uncertain. Perceived stress was assessed using a single item and dichotomized from its original four response categories, which may have resulted in loss of information and reduced measurement precision. In particular, reverse causality cannot be excluded because depressive symptoms and stress may themselves be associated with changes in appetite, eating behavior, physical activity, and consequently body weight. Thus, the observed associations may reflect bidirectional or shared underlying processes rather than an effect of weight change on mental health. Second, the temporal positioning of BMI and weight-control behavior warrants consideration. BMI was measured at the time of the KNHANES examination, after the 12-month period over which weight change was reported, while weight-control efforts were assessed over the same 12-month period. Therefore, these variables may not represent conventional confounders and could potentially lie on the causal pathway. Nevertheless, sensitivity analyses with and without adjustment for these variables yielded generally consistent results. Third, several limitations relate to the measurement of weight change. The KNHANES data did not allow us to clearly distinguish intentional from unintentional weight change. In particular, unintentional weight loss may reflect underlying physical illness or mental health problems, including depression, which could partly account for the observed association between weight loss and poorer mental health. In addition, weight change was assessed as an absolute change in kilograms rather than as a percentage of baseline body weight. Because baseline body weight was unavailable, we could not evaluate percentage weight change; thus, the same absolute weight change may have different clinical implications depending on an individual’s initial body weight.

Fourth, although additional sensitivity analyses adjusted for several clinical, behavioral, and socioeconomic factors, residual confounding by unmeasured or incompletely measured factors, such as other medical conditions, medication use, or family history, cannot be excluded. Fifth, the use of complete-case analysis may have introduced selection bias. Although 86.5% of eligible participants were retained and variable-specific missingness was relatively modest, participants excluded because of missing data differed from those included in the analytic sample on several sociodemographic and health-related characteristics. Therefore, the possibility that systematic differences associated with missingness influenced the observed associations cannot be excluded. Finally, cultural context must be acknowledged, as body weight attitudes differ across societies and may limit the generalizability of these findings beyond the Korean population.

Despite these limitations, this study contributes by incorporating weight-control behaviors and multiple measures of mental health in adulthood, thereby extending prior research that largely focused on associations between weight change and psychological distress (27) or focused on the association between weight gain and mental health outcomes (31). Our findings suggest that substantial weight change in either direction may serve as a marker warranting consideration of underlying physical and psychological factors. These findings should not be interpreted as indicating that weight change itself is harmful or that weight stability is necessarily protective, particularly when weight loss is clinically appropriate.

In conclusion, this study demonstrated that weight change was associated with poorer mental health in a nationally representative sample. Future research should employ longitudinal and culturally diverse designs, incorporate objective measures of stress and depression, and further examine the temporal and potentially bidirectional relationships between weight change and mental health.

## Data Availability

The original contributions presented in the study are included in the article/**Supplementary Material**, further inquiries can be directed to the corresponding author/s.
