# Supplementary material for: Associations of weight change with perceived stress and depression: a nationwide population-based study
Source: Front Psychiatry. 2026 Sep 16;17:1941947. doi: 10.3389/fpsyt.2026.1941947 (PMC13623869; doi:10.3389/fpsyt.2026.1941947)
Supplement: Supplementary file 1 [file SupplementaryFile1.docx]

**Supplementary Table S1**

**Missingness by study variable among eligible participants (N = 24,980)**

| Variable | Total N | Missing, n | Missing, % |
| --- | --- | --- | --- |
| Weight change | 24,980 | 1,743 | 6.98 |
| Depressive symptoms (PHQ-9) | 24,980 | 3,003 | 12.02 |
| Perceived stress | 24,980 | 1,740 | 6.97 |
| Self-reported physician diagnosis of depression | 24,980 | 2,694 | 10.78 |
| Sex | 24,980 | 0 | 0 |
| Age | 24,980 | 0 | 0 |
| Household income | 24,980 | 111 | 0.44 |
| Economic activity | 24,980 | 2,814 | 11.27 |
| Marital status | 24,980 | 9 | 0.04 |
| Living alone | 24,980 | 3 | 0.01 |
| Body mass index | 24,980 | 1,180 | 4.72 |
| Weight-control status | 24,980 | 1,709 | 6.84 |
| Survey year | 24,980 | 0 | 0 |
| Diabetes mellitus | 24,980 | 1,667 | 6.67 |
| Hypertension | 24,980 | 1,660 | 6.65 |
| Thyroid disease | 24,980 | 2,693 | 10.78 |
| Lifetime smoking | 24,980 | 1,731 | 6.93 |
| Lifetime alcohol use | 24,980 | 1,710 | 6.85 |
| Activity limitation | 24,980 | 2,723 | 10.9 |
| Education level | 24,980 | 2,822 | 11.3 |

**Supplementary Table S2**

**Characteristics of participants included in and excluded from the complete-case analysis**

| Characteristics | Excluded due to missing data (n=3,381) | Included in complete-case analysis (n=21,599) | P value |
| --- | --- | --- | --- |
| Age, years, mean | 51.72 | 46.71 | <0.001 |
| BMI, kg/m², mean | 23.95 | 23.99 | 0.666 |
| Sex, % |  |  | 0.074 |
| Male | 47.73 | 49.89 |  |
| Female | 52.27 | 50.11 |  |
| Age group, % |  |  | <0.001 |
| 19–35 years | 22.33 | 28.55 |  |
| 36–64 years | 48.07 | 55.88 |  |
| ≥65 years | 29.59 | 15.57 |  |
| Household income, % |  |  | <0.001 |
| Lowest quartile | 31.04 | 24.87 |  |
| Second quartile | 24.91 | 25.07 |  |
| Third quartile | 22.98 | 25.06 |  |
| Highest quartile | 21.07 | 25 |  |
| Economic activity, % |  |  | <0.001 |
| Economically active | 40.11 | 64.39 |  |
| Economically inactive | 59.89 | 35.61 |  |
| Living alone, % |  |  | 0.003 |
| Yes | 12.11 | 9.82 |  |
| No | 87.89 | 90.18 |  |
| Education level, % |  |  | <0.001 |
| Elementary school or less | 36.06 | 11.68 |  |
| Middle school | 7.94 | 8.55 |  |
| High school | 22.74 | 28.26 |  |
| College/university or higher | 33.26 | 51.51 |  |
| Diabetes mellitus, % |  |  | <0.001 |
| Yes | 12.74 | 7.56 |  |
| No | 87.26 | 92.44 |  |
| Hypertension, % |  |  | <0.001 |
| Yes | 26.01 | 17.81 |  |
| No | 73.99 | 82.19 |  |
| Thyroid disease, % |  |  | 0.344 |
| Yes | 4.31 | 3.39 |  |
| No | 95.69 | 96.61 |  |
| Lifetime smoking, % |  |  | 0.032 |
| Ever smoked | 40.27 | 43.38 |  |
| Never smoked | 59.73 | 56.62 |  |
| Lifetime alcohol use, % |  |  | <0.001 |
| Ever drank | 83.41 | 91.43 |  |
| Never drank | 16.59 | 8.57 |  |
| Activity limitation, % |  |  | <0.001 |
| Yes | 18.27 | 6.25 |  |
| No | 81.73 | 93.75 |  |

**Supplementary Table S3**

**Sensitivity analyses of associations between weight change and mental health outcomes with and without adjustment for BMI and weight-control status**

**A. Depressive symptoms (PHQ-9)**

| Weight change | Model 1 | Model 2 | Model 3 | Model 4 |
| --- | --- | --- | --- | --- |
| No weight change | 1.00 (Ref.) | 1.00 (Ref.) | 1.00 (Ref.) | 1.00 (Ref.) |
| Loss cat 1 | 3.51 (2.30–5.34) | 3.60 (2.36–5.49) | 3.74 (2.46–5.69) | 3.80 (2.49–5.79) |
| Loss cat 2 | 1.57 (1.01–2.42) | 1.59 (1.03–2.46) | 1.62 (1.05–2.50) | 1.64 (1.06–2.53) |
| Loss cat 3 | 1.72 (1.37–2.16) | 1.73 (1.38–2.17) | 1.78 (1.41–2.24) | 1.78 (1.42–2.24) |
| Gain cat 1 | 1.36 (1.12–1.65) | 1.40 (1.14–1.71) | 1.41 (1.15–1.71) | 1.43 (1.17–1.75) |
| Gain cat 2 | 2.27 (1.70–3.03) | 2.42 (1.79–3.27) | 2.33 (1.74–3.11) | 2.44 (1.80–3.30) |
| Gain cat 3 | 3.82 (2.72–5.36) | 4.23 (2.90–6.17) | 3.96 (2.82–5.56) | 4.26 (2.92–6.23) |

**B. Self-reported physician diagnosis of depression**

| Weight change | Model 1 | Model 2 | Model 3 | Model 4 |
| --- | --- | --- | --- | --- |
| No weight change | 1.00 (Ref.) | 1.00 (Ref.) | 1.00 (Ref.) | 1.00 (Ref.) |
| Loss cat 1 | 1.99 (1.17–3.38) | 1.99 (1.17–3.38) | 2.01 (1.18–3.42) | 2.01 (1.19–3.42) |
| Loss cat 2 | 2.07 (1.23–3.47) | 2.07 (1.24–3.47) | 2.08 (1.24–3.50) | 2.08 (1.24–3.50) |
| Loss cat 3 | 1.23 (0.96–1.58) | 1.23 (0.96–1.59) | 1.24 (0.97–1.59) | 1.24 (0.97–1.59) |
| Gain cat 1 | 1.50 (1.23–1.83) | 1.51 (1.23–1.85) | 1.51 (1.24–1.84) | 1.51 (1.24–1.85) |
| Gain cat 2 | 1.97 (1.39–2.79) | 1.98 (1.39–2.83) | 1.98 (1.40–2.80) | 1.99 (1.39–2.84) |
| Gain cat 3 | 2.80 (1.89–4.14) | 2.83 (1.86–4.31) | 2.82 (1.90–4.17) | 2.84 (1.86–4.32) |

**C. Perceived stress**

| Weight change | Model 1 | Model 2 | Model 3 | Model 4 |
| --- | --- | --- | --- | --- |
| No weight change | 1.00 (Ref.) | 1.00 (Ref.) | 1.00 (Ref.) | 1.00 (Ref.) |
| Loss cat 1 | 1.83 (1.34–2.48) | 1.82 (1.34–2.48) | 1.85 (1.37–2.52) | 1.85 (1.36–2.51) |
| Loss cat 2 | 1.37 (1.09–1.72) | 1.37 (1.09–1.72) | 1.39 (1.11–1.74) | 1.39 (1.10–1.74) |
| Loss cat 3 | 1.38 (1.22–1.56) | 1.38 (1.22–1.56) | 1.39 (1.23–1.57) | 1.39 (1.23–1.57) |
| Gain cat 1 | 1.28 (1.16–1.41) | 1.27 (1.15–1.41) | 1.29 (1.16–1.42) | 1.28 (1.16–1.42) |
| Gain cat 2 | 1.52 (1.29–1.78) | 1.51 (1.28–1.78) | 1.52 (1.30–1.79) | 1.51 (1.28–1.78) |
| Gain cat 3 | 2.28 (1.82–2.85) | 2.26 (1.79–2.87) | 2.30 (1.84–2.87) | 2.26 (1.79–2.86) |

***Note****.* No weight change was the reference category. Loss categories 1, 2, and 3 indicate ≥10 kg loss, 6–<10 kg loss, and 3–<6 kg loss, respectively. Gain categories 1, 2, and 3 indicate 3–<6 kg gain, 6–<10 kg gain, and ≥10 kg gain, respectively. All models were adjusted for sex, age, income level, employment status, marital status, and living arrangement. Model 1 did not include BMI or weight-control status. Model 2 was additionally adjusted for BMI, Model 3 for weight-control status, and Model 4 for both BMI and weight-control status.

**Supplementary Table S4**

**Sensitivity analyses with additional adjustment for clinical and behavioral covariates**

**A. Depressive symptoms (PHQ-9)**

| Weight change | Base-adjusted model | Additionally adjusted model |
| --- | --- | --- |
| No weight change | 1.00 (Ref.) | 1.00 (Ref.) |
| Loss cat 1 | 3.80 (2.49–5.79) | 3.08 (1.96–4.84) |
| Loss cat 2 | 1.64 (1.06–2.54) | 1.32 (0.82–2.11) |
| Loss cat 3 | 1.78 (1.42–2.24) | 1.52 (1.20–1.94) |
| Gain cat 1 | 1.43 (1.17–1.75) | 1.41 (1.15–1.74) |
| Gain cat 2 | 2.44 (1.80–3.30) | 2.29 (1.68–3.12) |
| Gain cat 3 | 4.26 (2.92–6.23) | 3.84 (2.61–5.65) |

**B. Self-reported physician diagnosis of depression**

| Weight change | Base-adjusted model | Additionally adjusted model |
| --- | --- | --- |
| No weight change | 1.00 (Ref.) | 1.00 (Ref.) |
| Loss cat 1 | 2.01 (1.18–3.41) | 1.70 (1.01–2.85) |
| Loss cat 2 | 2.02 (1.19–3.42) | 1.79 (1.03–3.14) |
| Loss cat 3 | 1.24 (0.97–1.59) | 1.11 (0.86–1.43) |
| Gain cat 1 | 1.51 (1.23–1.85) | 1.48 (1.21–1.82) |
| Gain cat 2 | 1.99 (1.39–2.84) | 1.79 (1.24–2.60) |
| Gain cat 3 | 2.83 (1.86–4.32) | 2.42 (1.58–3.71) |

**C. Perceived stress**

| Weight change | Base-adjusted model | Additionally adjusted model |
| --- | --- | --- |
| No weight change | 1.00 (Ref.) | 1.00 (Ref.) |
| Loss cat 1 | 1.85 (1.36–2.51) | 1.72 (1.25–2.35) |
| Loss cat 2 | 1.38 (1.09–1.73) | 1.29 (1.03–1.63) |
| Loss cat 3 | 1.39 (1.23–1.57) | 1.34 (1.18–1.51) |
| Gain cat 1 | 1.28 (1.16–1.41) | 1.26 (1.14–1.39) |
| Gain cat 2 | 1.51 (1.28–1.78) | 1.41 (1.20–1.67) |
| Gain cat 3 | 2.26 (1.79–2.86) | 2.08 (1.65–2.62) |

***Note***. No weight change was the reference category. Loss categories 1, 2, and 3 indicate ≥10 kg loss, 6–<10 kg loss, and 3–<6 kg loss, respectively. Gain categories 1, 2, and 3 indicate 3–<6 kg gain, 6–<10 kg gain, and ≥10 kg gain, respectively. The base-adjusted model was adjusted for sex, age, income level, employment status, marital status, living arrangement, BMI, and weight-control status. The additionally adjusted model was further adjusted for diabetes, hypertension, thyroid disease, smoking, alcohol use, activity limitation, and education level. Both models were estimated using the same analytic sample.

**Supplementary Table S5**

**Sensitivity analyses accounting for survey year and exclusion of the 2020 survey cycle**

**A. Depressive symptoms (PHQ-9)**

| Weight change | Model 1 | Model 2 | Model 3 |
| --- | --- | --- | --- |
| No weight change | 1.00 (Ref.) | 1.00 (Ref.) | 1.00 (Ref.) |
| Loss cat 1 | 3.80 (2.49–5.79) | 3.82 (2.50–5.85) | 4.21 (2.54–6.99) |
| Loss cat 2 | 1.64 (1.06–2.53) | 1.64 (1.06–2.54) | 1.86 (1.14–3.02) |
| Loss cat 3 | 1.78 (1.42–2.24) | 1.76 (1.40–2.22) | 1.70 (1.32–2.20) |
| Gain cat 1 | 1.43 (1.17–1.75) | 1.44 (1.18–1.76) | 1.34 (1.06–1.69) |
| Gain cat 2 | 2.44 (1.80–3.30) | 2.44 (1.81–3.31) | 2.48 (1.75–3.50) |
| Gain cat 3 | 4.26 (2.92–6.23) | 4.25 (2.90–6.23) | 3.71 (2.40–5.72) |

**B. Self-reported physician diagnosis of depression**

| Weight change | Model 1 | Model 2 | Model 3 |
| --- | --- | --- | --- |
| No weight change | 1.00 (Ref.) | 1.00 (Ref.) | 1.00 (Ref.) |
| Loss cat 1 | 2.01 (1.19–3.42) | 2.00 (1.18–3.40) | 1.59 (0.78–3.23) |
| Loss cat 2 | 2.08 (1.24–3.50) | 2.09 (1.24–3.51) | 2.55 (1.42–4.55) |
| Loss cat 3 | 1.24 (0.97–1.59) | 1.23 (0.96–1.58) | 1.24 (0.92–1.68) |
| Gain cat 1 | 1.51 (1.24–1.85) | 1.52 (1.24–1.86) | 1.53 (1.20–1.94) |
| Gain cat 2 | 1.99 (1.39–2.84) | 1.99 (1.39–2.84) | 1.79 (1.14–2.81) |
| Gain cat 3 | 2.84 (1.86–4.32) | 2.83 (1.86–4.30) | 2.55 (1.59–4.08) |

**C. Perceived stress**

| Weight change | Model 1 | Model 2 | Model 3 |
| --- | --- | --- | --- |
| No weight change | 1.00 (Ref.) | 1.00 (Ref.) | 1.00 (Ref.) |
| Loss cat 1 | 1.85 (1.36–2.51) | 1.83 (1.35–2.50) | 1.84 (1.24–2.73) |
| Loss cat 2 | 1.39 (1.10–1.74) | 1.38 (1.10–1.74) | 1.57 (1.21–2.04) |
| Loss cat 3 | 1.39 (1.23–1.57) | 1.40 (1.24–1.58) | 1.35 (1.18–1.54) |
| Gain cat 1 | 1.28 (1.16–1.42) | 1.27 (1.15–1.41) | 1.22 (1.08–1.37) |
| Gain cat 2 | 1.51 (1.28–1.78) | 1.50 (1.28–1.77) | 1.40 (1.15–1.71) |
| Gain cat 3 | 2.26 (1.79–2.86) | 2.26 (1.79–2.86) | 2.03 (1.53–2.68) |

***Note***. No weight change was the reference category. Loss categories 1, 2, and 3 indicate ≥10 kg loss, 6–<10 kg loss, and 3–<6 kg loss, respectively. Gain categories 1, 2, and 3 indicate 3–<6 kg gain, 6–<10 kg gain, and ≥10 kg gain, respectively. Model 1 was adjusted for sex, age, income, employment, marital status, living arrangement, BMI, and weight-control status; Model 2 additionally included categorical survey year; and Model 3 replicated Model 2 after excluding the 2020 survey cycle.
